# Nanostructured TiO_2_ anatase-rutile-carbon solid coating with visible light antimicrobial activity

**DOI:** 10.1038/s41598-018-38291-y

**Published:** 2019-02-13

**Authors:** Susan P. Krumdieck, Raphaël Boichot, Rukmini Gorthy, Johann G. Land, Sabine Lay, Aleksandra J. Gardecka, Matthew I. J. Polson, Alibe Wasa, Jack E. Aitken, Jack A. Heinemann, Gilles Renou, Grégory Berthomé, Frédéric Charlot, Thierry Encinas, Muriel Braccini, Catherine M. Bishop

**Affiliations:** 1 Advanced Energy and Material Systems Laboratory, Department of Mechanical Engineering, University of Canterbury, Christchurch, 8041 New Zealand; 2 Univ. Grenoble Alpes, CNRS, Grenoble INP (Institute of Engineering), SIMAP, F-38000 Grenoble, France; 3 Department of Chemistry, University of Canterbury, Christchurch, 8041 New Zealand; 4 School of Biological Sciences, University of Canterbury, Christchurch, 8041 New Zealand

## Abstract

TiO_2_ photocatalyst is of interest for antimicrobial coatings on hospital touch-surfaces. Recent research has focused on visible spectrum enhancement of photocatalytic activity. Here, we report TiO_2_ with a high degree of nanostructure, deposited on stainless steel as a solid layer more than 10 μm thick by pulsed-pressure-MOCVD. The TiO_2_ coating exhibits a rarely-reported microstructure comprising anatase and rutile in a composite with amorphous carbon. Columnar anatase single crystals are segmented into 15–20 nm thick plates, resulting in a *mille-feuilles* nanostructure. Polycrystalline rutile columns exhibit dendrite generation resembling pine tree *strobili*. We propose that high growth rate and co-deposition of carbon contribute to formation of the unique nanostructures. High vapor flux produces step-edge instabilities in the TiO_2_, and solid carbon preferentially co-deposits on certain high energy facets. The equivalent effective surface area of the nanostructured coating is estimated to be 100 times higher than standard TiO_2_ coatings and powders. The coatings prepared on stainless steel showed greater than 3-log reduction in viable *E coli* after 4 hours visible light exposure. The pp-MOCVD approach could represent an up-scalable manufacturing route for supported catalysts of functional nanostructured materials without having to make nanoparticles.

## Introduction

Antimicrobial touch surface materials are of high interest in response to concerns about antibiotic resistance and the growing crisis of nosocomial infections^1–3^. TiO_2_ has received much attention as a possible antimicrobial material due to its well-known photocatalytic activity (PCA) and self-cleaning properties^4^. TiO_2_ has selective spectral absorption in the ultraviolet region but is transparent to visible light. Absorbed UV photons with energies greater than 3.2 eV for anatase and 3.0 eV for rutile generate electron-hole pairs that can migrate to a crystal surface where redox reactions occur^5^. Reactive oxygen species (ROS) are generated by reduction and oxidation of water or oxygen. ROS are non-toxic, and continuously regenerated on the surface, providing a highly desirable approach for diminishing the viability of most microorganisms on touch surfaces^6^.

TiO_2_ photocatalytic properties have been known for many years^4^ but enhancing the PCA for antimicrobial applications under visible light remains a challenge. The strategies for PCA enhancement are to increase surface area, reduce carrier migration path length, select for more active facets and extend the band gap into the visible spectrum^7^. Much of the research interest has been focused on nanoparticles, which have higher specific surface area compared to bulk phases and shorter exciton migration path to an active surface^8^. Crystal thickness less than 15 nm have exhibited higher PCA than polycrystals due to reduced recombination of electrons and holes at internal crystal defects^9,10^.

Anatase nanocrystals with prominent high-energy facets, e.g. {001} and {10 *l*}, have been nanoengineered using fluorine-termination in hydrothermal processes^11,12^. Rutile has a smaller bandgap than anatase, but rutile is more challenging to produce as nanoparticles^13^. Rutile-anatase heterojunctions have demonstrated higher PCA than either single phase material^14^. The proposed mechanism for enhanced PCA of mixed-phase TiO_2_ is a type-II, staggered valence and conduction band alignment at the interface favorable to separation of charge carriers^15^.

Enhanced PCA has also been reported using mixtures of TiO_2_ powders with activated carbon, graphene and carbon nanotubes^16–18^. Higher PCA of carbonaceous materials has been attributed to two main functions of carbon on the TiO_2_ surface. Carbon acts as a photosensitizer, effectively extending the excitation wavelength and tuning the band-gap. Carbon also promotes the surface reactions by providing higher adsorption of organic molecules at active sites^19^.

TiO_2_ is non-toxic, but the antimicrobial efficacy is directly related to the PCA because the radical oxygen species are extremely toxic and acutely lethal^20^. There are several challenges that must be overcome in order to manufacture a coating on door handles or bed rails for health care facilities:

- Producing a nanoengineered material with high specific surface area, in a solid robust coating.
- Extending the PCA, and thus antimicrobial efficacy, into the visible spectrum.
- Developing an up-scalable process for coatings on stainless steel.

Nearly all photocatalytic TiO_2_ studies reported in the literature involve nanoparticles synthesized by hydrothermal processes^21^. Coating 3-D surfaces typically requires an epoxy binder or paint mixture with suspended TiO_2_ nanoparticles. Paints have wear-related issues on touch surfaces, and the TiO_2_ would be encased in the binder, reducing the effective surface area for antimicrobial activity. It would also be difficult to form a polymer coating which would not be degraded by the ROS produced by photocatalysis.

The up-scalable pulsed-pressure metalorganic chemical vapor deposition (pp-MOCVD) process used in this work has been described in detail elsewhere^22^. The pp-MOCVD process uses flash vaporization of liquid precursor, and deposition occurs during periodic sharp spikes in vapor pressure^23^. We have previously reported the emergence of fine columnar morphology when the substrate temperature is high enough for pyrolytic carbon deposition and mass transport-controlled growth^24^.

In this work, we investigate a deposition regime with very high instantaneous precursor vapor flux, ten times higher than our previous work. The high pulsed growth rate produces a TiO_2_ coating material that is a composite of nanostructured anatase, rutile dendrites and carbon (NsARC). NsARC has a remarkable micro-structure, nanoscale single crystallinity and strong adhesion on stainless steel. TiO_2_ thin films with similar morphologies have been reported by only a few research groups^25–27^, but none of them included PCA or antimicrobial testing. Additionally, in these papers the deposition rates were not given, none of the processes were up-scalable, and the presence of carbon in the coating has not been reported. Here, we report thick, robust and adherent NsARC coating on stainless steel which exhibits high antimicrobial activity under visible light. We present the first demonstration of a carbonaceous TiO_2_ coating material that combines the key PCA enhancement strategies of rutile-anatase heterojunctions, nanostructured single crystals with high surface area and low migration path length.

## Results

**Figure 1:**
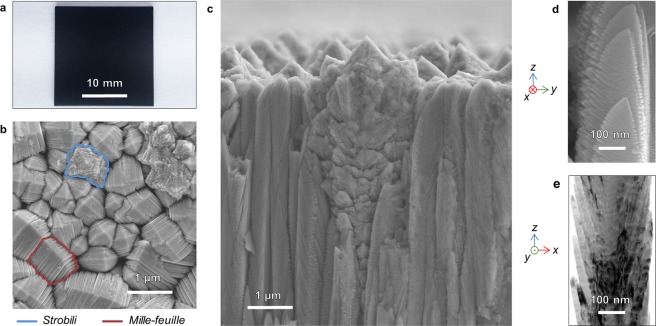
As deposited NsARC TiO_2_. (**a**) Stainless-steel substrate with 10 μm thick coating. (**b**) Scanning Electron Microscope (SEM) image of coating surface morphology with *mille-feuilles* and *strobili* structures indicated. (**c**) SEM fracture surface cross-section showing *mille-feuilles* columns and *strobili* dendrites both with strong z-orientation. (**d**) High magnification SEM image of side-view of a *mille-feuilles* column. (**e**) Bright field TEM image of a *mille-feuilles* column, probably rotated 90° relative to (**d**).

The TiO_2_ coating described in this work has been produced repeatably on more than 200 samples. Typical processing time of 60 minutes produces 10 μm thick films which exhibit remarkable adhesion and durability (Supplementary Fig. S1). The material appears uniform and deep black in color on both fused silica and stainless-steel substrates, providing unambiguous evidence of visible light absorption (Fig. 1a). When annealed at 500 °C for 2 hours in air, the NsARC film becomes white without any other observed changes in adhesion or microstructure. Highly disordered TiO_2_ nanocrystals and Ti_2_O_3_ have also been reported to be black^28^. Raman analysis of NsARC before and after annealing indicates that solid carbon in the material, rather than disordered TiO_2_, is responsible for the visible light absorption and black color (Supplementary Fig. S2).

### Morphology and Nanostructure

The NsARC surface has two distinct morphologies: (Fig. 1b).

*mille-feuilles -* slightly asymmetric pyramidal structures composed of multiple layers.

*strobili* - branched dendrite structures resembling pinecones.

The fractured surface cross-section shows that the *mille-feuilles* are fine needle-like columns that extend through the entire thickness of the film (Fig. 1c). The nanostructure of the *mille-feuilles* is composed of thin plates oriented close to parallel to the growth direction and exhibiting further branching along the edges with a feather-like appearance (Fig. 1d). Transmission Electron Microscopy (TEM) imaging of scraped-off NsARC shows the segmentation of the columnar crystals into plates (Fig. 1e). The *mille-feuilles* nanoplates are 10–20 nm in thickness. Selected Area Electron Diffraction (SAED) analysis shows that the plates making up a column, and the core from which they extend, are all anatase with the same orientation, and with no discernible grain boundaries within the plates (Supplementary Fig. S3). The length of the exciton migration path in these anatase plate structures would be less than 15 nm to an active surface.

The *strobili* exhibit dendritic growth with secondary branches growing from a columnar core (Fig. 1c). The surface view (Fig. 1b) shows the *strobili* petals radiating from the main core which normally has a primary axis. The thickness of the petals is less than 20 nm, again providing nano-scale features for PCA enhancement (Supplementary Fig. S3). There were no identifiable pieces of in-tact *strobili* found in the TEM examinations of scraped-off coatings.

### Crystallographic Characterization

**Figure 2:**
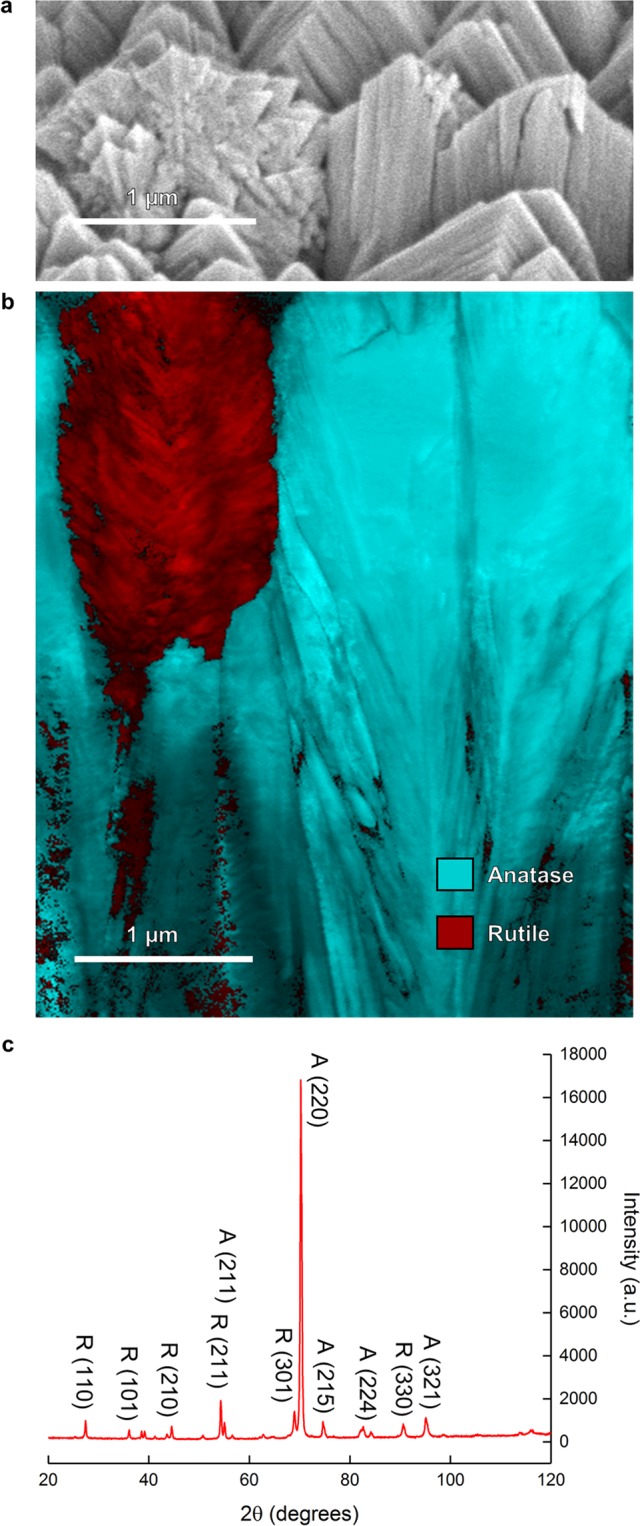
Phase Characterization. (**a**) Surface SEM image showing one *strobili* and two *mille-feuilles* columns extracted by FIB. (**b**) ACOM phase analysis of the extracted columns, definitively identifying the *mille-feuilles* structures as anatase and the *strobili* structures as rutile. (**c**) XRD spectrum showing (220) texture of anatase and the presence of rutile.

The material contains both rutile and anatase phases of TiO_2_ according to XRD analysis (Fig. 2c). The one *strobili* and two *mille-feuilles* structures shown in surface view SEM (Fig. 2a) were sectioned using a Focused Ion Beam (FIB). The internal structure of the two morphologies is quite different (Fig. 2b). Automated Crystal Orientation and Phase Mapping (ACOM)^29^ identified the *mille-feuilles* as anatase and the *strobili* as rutile (Fig. 2b). The ACOM results show that the *strobili* are phase-pure rutile, and the *mille-feuilles* are highly oriented, single crystal anatase.

The anatase phase has a rarely reported (220) texture (Fig. 2c). This is interesting because the segmented *mille-feuilles* plates observed in Fig. 2a align with the {001} planes. The anatase {001} facets have been identified in hydro-thermally produced nanoparticles as having enhanced PCA due to “surface heterojunctions” with {101} facets^11^. The plates observed along the length of the anatase columns appear to be inclined along {10 *l*} facets. The facets on the anatase columns determined from angles measured in SEM images relative to the substrate normal are {107}, which has not been described before in the literature.

A *strobili* microstructure had been identified as rutile by Takahashi *et al*.^25^ by deduction. Takahashi’s thin films had only anatase according to XRD and had no visible *strobili*. Thicker films had visible *strobili* morphologies, and XRD spectra showed rutile as well as anatase. NsARC cross section SEM images show the *strobili* columns growing from very near the substrate surface. Rutile *Strobili* are randomly dispersed among majority anatase *mille-feuilles* (Fig. 1b). The phenomena responsible for initiation of anatase vs. rutile phases is unclear, but the deposition conditions strongly favor columnar growth of recognizably distinctive morphologies.

### Compositional Characterization

**Figure 3:**
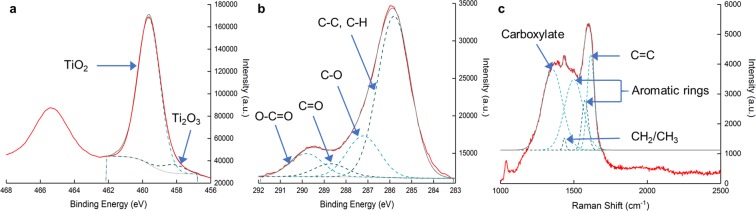
Composition Characterization. (**a**) XPS spectrum in the range for titanium oxides with peak deconvolution showing TiO_2_ and Ti_2_O_3_. (**b**) XPS spectrum for the range for carbon and carbon compounds with peak deconvolution. (**c**) Raman spectrum with peak deconvolution for carbon organic structures.

XPS analysis of the NsARC coating shows the characteristic peaks for TiO_2_ at binding energies of 459.7 and 465.4 eV (Fig. 3a). Optimized peak fitting revealed a small signal from Ti_2_O_3_ that is attributed to ion-induced damage from the cleaning process to remove adventitious carbon^27^. There was no Ti_2_O_3_ detected in the XRD analysis or identified by TEM-SAD. Carboxylic groups were identified in the 283–292 eV range of XPS, but no detectable Ti-C bonds are observed, which indicates no carbon doping of the TiO_2_ (Fig. 3b).

The spectrum of carbon in the film consists of a composite of peaks between 283 eV and 292 eV (Fig. 3c). The strongest signal at 285.8 eV indicates the presence of C-C or C-H bonds in the films. The other peak signals at 287.2 eV, 288.6 eV and 289.8 eV are the signatures of C-O, C=O and O-C=O bonds with lower intensities.

The surface enhanced Raman spectroscopy identified amorphous carbon in the material (Supplementary Fig. S2), mostly in the form of aromatic rings and carboxylate groups (Fig. 3c). In a previous study of TiO_2_ thin films on FTO substrate, the depth-profile XPS indicated that the carbon was present in the same form at all depths of the film^30^. It is extremely difficult to directly observe carbon with SEM, TEM, atomic force microscope (AFM) or other related imaging techniques. Given the evidence presented here, we propose that the carbon is located in the areas that appear to be spaces in the SEM and TEM images. These spaces between *mille-feuilles* plates and *strobili* surfaces are observed throughout the depth of the material.

### Phase Morphologies

**Figure 4:**
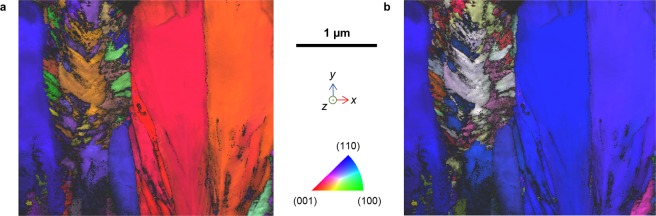
ACOM orientation mapping. The anatase *mille-feuilles* columns are highly oriented single crystals and rutile *strobili* are polycrystalline. (**a**) Projection parallel to substrate and (**b**) Projection in the growth direction normal to the substrate surface.

The orientations of the crystals in the FIB sample were observed with ACOM orientation mapping analysis (Fig. 4). Both projections show that the anatase columns are single crystals and the rutile columns are composed of many small single crystals that have apparently random orientations. The first projection of crystal planes parallel to the substrate surface (Fig. 4a) indicates that the three visible anatase columns have slightly different in-plane rotational alignments, consistent with the surface SEM observation (Fig. 2a). The second projection in the growth direction clearly shows that all of the anatase columns have (110) orientation, nearly parallel to the substrate and consistent with the strong (220) texture found in the XRD spectra.

### Carbon Locus Investigation

**Figure 5:**
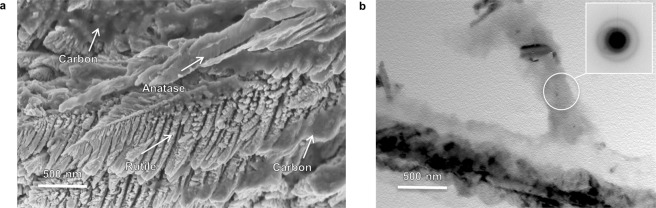
Carbon locus investigation. Acid dissolution of a sample of NsARC removes most of the anatase columns and reveals the internal structure of rutile columns. (**a**) SEM image of rutile *strobili* skeleton and remaining anatase skeletons showing amorphous carbon deposits throughout the material. (**b**) TEM image of rutile skeleton coated by amorphous carbon identified by SAED (inset).

The location of the amorphous carbon was further investigated by acid dissolution (HF 50%, 24 hours). Acid etching preferentially attacks anatase over rutile and does not dissolve carbon^31^. Amorphous carbon on the TiO_2_ facet surfaces should consolidate into a tar residue as the TiO_2_ is removed. Observation of the locations of residue could indicate whether carbon is equally deposited on rutile and anatase. The etched structure shows the remaining dendritic skeleton of the rutile *strobili* (Fig. 5a). Smooth deposits on the remains of anatase columns are also interpreted as carbon residue. TEM with SAED analysis of the etched sample shows dense regions of amorphous carbon surrounding the remaining TiO_2_ crystals (Fig. 5b). The etched materials provide evidence that the carbon is present on both phases, but it is still unclear if the carbon is preferentially co-deposited on certain preferred facets, or if it is equally deposited on all exposed facets.

### Pyrolytic Carbon Co-Deposition

Nearly all studies of TiO_2_ thin films report adventitious carbon contamination on the free surfaces exposed to air^32^. However, there have been no previous reports of pyrolytic carbon co-deposition on crystal surfaces throughout the thickness of TiO_2_ deposited by MOCVD from TTIP precursor. The pp-MOCVD process in this work is not supplied with O_2_ reactant gas or water vapor. FACTSAGE 7.1 equilibrium analysis^33^ at the deposition temperature and peak pulse pressure indicates that solid carbon would be a product of pyrolytic reactions (Supplementary Fig. S4). The pulsed pressure processing results in conditions far away from equilibrium, but the analysis gives a clear indication that the pyrolysis of the TTIP and toluene vapor mixture could lead to graphitic carbon formation. The catalytic effects of the titania surfaces could also locally favor the carbon formation. It is reasonable to assume that carbon is co-deposited with the TiO_2_ during each deposition pulse. Carbon is thermodynamically stable at the deposition temperature in vacuum, so once deposited on a surface it would remain in place. Thus, we conclude that pyrolytic carbon is co-deposited on the TiO_2_ surfaces and facets throughout the material.

### Proposed Mechanisms for Growth of NsARC Nanostructures

**Figure 6:**
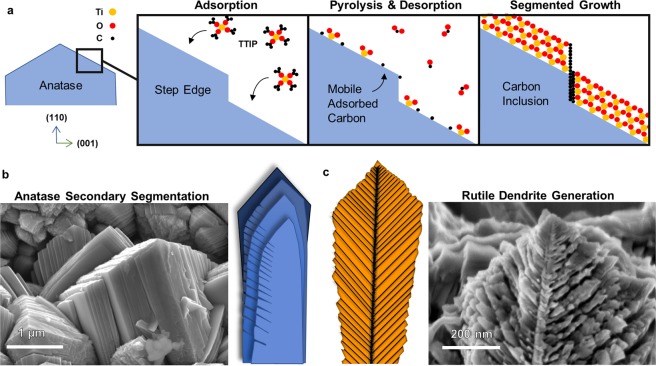
Proposed mechanisms of nanostructure development. (**a**) High flux rate of precursor vapor produces rapid growth of anatase crystals with unstable periodic step edges which become capped with carbon and result in segmented plate nanostructure. (**b**) SEM image of a *mille-feuille* anatase column top with primary segmentation and illustration of secondary segmentations. (**c**) Rapid growth causes dendrite generation in rutile crystals as illustrated in the SEM image of an HF etched *strobili* dendrite.

Here we propose a mechanism by which the combination of high mass flux, fast pyrolysis and solid carbon formation produce the unique *mille-feuille* structure (Fig. 6a). The overall deposition rate was 15 nm/pulse. Most of the TTIP is assumed to react on the surface within about 0.34 sec during the peak vapor pulse (Supplementary Fig. S4). This means that the effective growth rate is around 160 μm/h. High crystal growth rate has been shown to cause unstable step edges to emerge in growing TiO_2_^34^.

Carbon may be catalytically reduced from TTIP ligands at step edges. Solid adsorbed pyrolytic carbon is expected to have sufficient diffusivity on the growing TiO_2_ crystal surfaces to migrate to preferential sites. If the carbon selectively accumulates at the step edge, then it could effectively terminate a crystal plane which would otherwise not be stable. The accumulated carbon thus causes segmented growth along these facets. These carbon-stabilized planes form the blueprint of the anatase nanostructure of segmented plates and gives rise to the feather-like features along the edges (Fig. 6b). Selective surface carbon capping on certain facets has been reported in hydrothermal processing using carboxylic terminated molecules, but has not been reported in vapor deposition^35,36^.

The internal dendritic structure of the rutile *strobili* columns can be seen in the crystal etched in 50% HF solution (Fig. 6c). The *strobili* exhibit dendritic formation from a central core, with primary branches of similar thickness from the base to the top. The trunks of the *strobili* are normal to the substrate surface, but the branches and dendrite generation create polycrystals with different orientations as already observed (Fig. 4). The carbon may have some role in the degree of dendrite generation, but the structure could also be produced by instabilities due to the high growth rate.

### Specific Surface Area Enhancement

**Figure 7:**
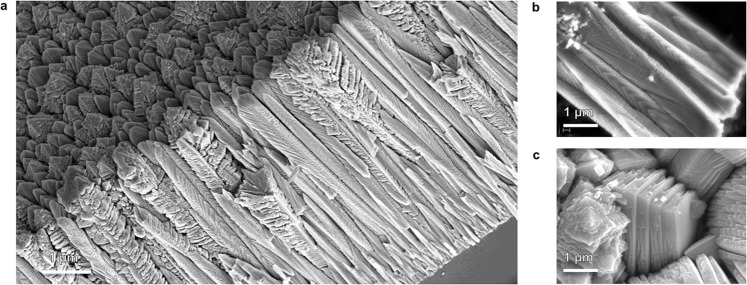
SEM micrograph of NsARC morphology showing high specific surface area. (**a**) Fracture surface showing crystals extending the entire depth of the coating and showing porosity between crystals. (**b**) Scraped off anatase *mille-feuilles* crystals showing the nanoplate structure. (**c**) Plan view SEM image showing the porosity between columns and between *mille-feuilles* plates.

High specific surface area is an extremely interesting consequence of the segmented anatase and dendritic rutile nanostructures. Macroscopically, the coating material behaves as a solid, fully dense coating. Microscopically, the material has porosity observed as voids between columnar crystals, plates and feathers (Fig. 7a). At the nanoscale, the space between the *mille-feuilles* segmented plates is observed to be open to ingress of water vapor or liquid water (Fig. 7b), and thus provides active surface area to produce ROS for photocatalysis or antimicrobial activity. The rutile *strobili* branches also appear to have open spaces between them.

We estimate that 10 µm NsARC films have specific surface area 80–140 times higher than a flat, non-porous TiO_2_ surface (see calculation in supplementary section). Most of this surface area appears to be available for surface reactions due to porous pathways from openings in the uppermost surface (Fig. 7c). In an aqueous environment, this means that the ROS generated throughout the material can diffuse into the solution and are available to degrade proteins and other organic molecules.

### Antimicrobial Performance

**Figure 8:**
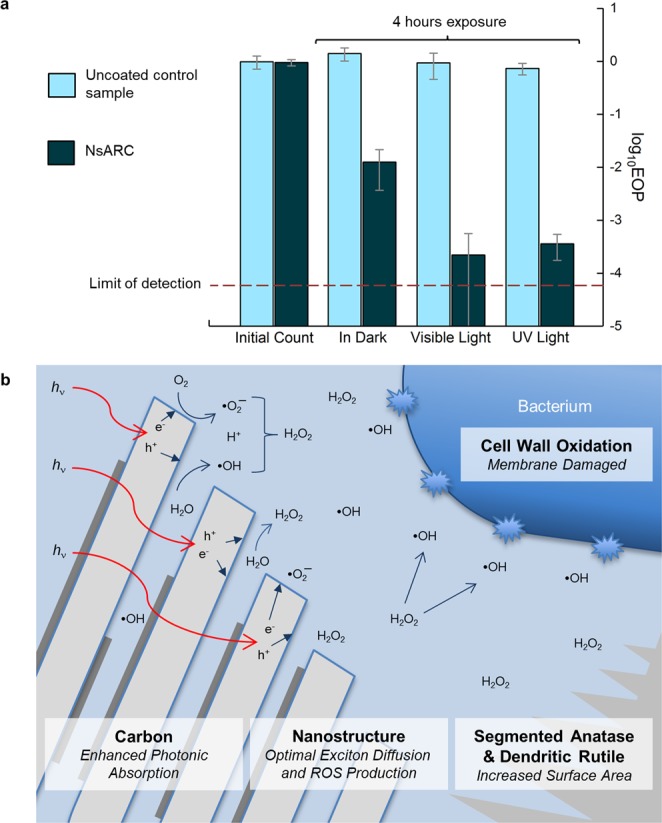
Antimicrobial performance of NsARC. (**a**) Antimicrobial results showing 99.9% to 99.99% reduction in viable *E coli* bacteria on NsARC coated stainless steel after four hours. (**b**) Mechanisms of AMA: photonic light absorption by carbon and TiO_2_, diffusion of electrons and holes, surface reactions with water and oxygen to form ROS, diffusion of ROS through the aqueous environment, and damage of bacterium cell membrane.

Efficacy under artificial indoor light is a key requirement for antimicrobial coating applications. In the health care environment, visible light is provided by commercial lamps at about 1000–1500 lumens. The antimicrobial activity (AMA) of NsARC on stainless steel was assessed using the international standard ISO 27447:2009. The efficiency of plating (EOP) is the number of *E. coli* live cells on NsARC compared to the uncoated sample under the same conditions. The number of viable bacteria decreased at least 1000-fold (99.9% reduction) after the 4-hour UV exposure. Remarkably, 3–4 log AMA was also achieved under visible light exposure (Fig. 8a).

Killing higher than 2 log AMA under visible light is difficult to achieve on TiO_2_ without elemental doping, for example with Mn^37^, or addition of biocidal copper or silver particles^38^. Interestingly, the NsARC coatings also had 2 log AMA even in the dark, which is normally only possible for toxic chemicals or metals^39^. Aqueous formate solution with rutile and anatase powders has been shown to generate carbon-centered radicals and ROS that can linger after irradiation to provide some bacterial growth suppression in the dark^40^. The AMA in the dark could also result from the NsARC coating modifying the zeta potential of bacteria cells by contact and changing the permeability of the membrane^41^ (Supplemental Fig. S5).

Interestingly, the NsARC coatings also had 2 log AMA even in the dark, which is normally only possible for toxic chemicals or metals^39^. Aqueous formate solution with rutile and anatase powders has been shown to generate carbon-centered radicals and ROS that can linger after irradiation to provide some bacterial growth suppression in the dark^40^. The AMA in the dark could also result from the NsARC coating modifying the zeta potential of bacteria cells by contact and changing the permeability of the membrane^41^ (Supplemental Fig. S5).

## Summary and Discussion

We set out to use the well-known pp-MOCVD process to prepare TiO_2_ coatings on stainless steel for antimicrobial touch surfaces applications. The objective was a one-step processing route for a robust solid coating of carbonaceous rutile-anatase heterojunctions and high specific surface area. The pp-MOCVD process can produce a wide range of vapor arrival rate, depending on the precursor concentration and injection volume^42^. The pp-MOCVD coatings are strongly adherent and uniform over 25 mm × 75 mm substrates and are good candidates for up-scaling to touch-surface products^22^. We used a much higher growth rate and discovered a remarkably black material with rarely reported morphologies. In this paper we have revealed the striking nanostructure and composition of a new NsARC TiO_2_ coating material, which has a combination of desired attributes for enhanced antimicrobial activity (Fig. 8b). Antimicrobial testing under visible light for the relatively short period of 4 hours demonstrated greatly enhanced lethality compared to other TiO_2_ studies in the literature.

The 10 μm thick NsARC coatings are comprised of anatase *mille-feuilles* and rutile *strobili* columnar structures in a composite with amorphous carbon. The anatase has nanoscale segmented single crystals and the rutile has high dendrite generation of nanoscale branches. The segmentation and dendrite generation extend the entire depth of the coating and the resulting extraordinarily high specific surface area is accessible to air and water, allowing generated ROS to diffuse from internal surface areas to the coating surface. We have proposed that very high crystal growth rate in the pp-MOCVD process generates step edges in the fast-growing crystals. We further propose that the unique pulsed-pressure processing results in solid carbon co-deposition, which caps high-energy crystal facets giving rise to the segmented nanostructure. The incorporation of carbon on the crystal facets extends the light adsorption into the visible range. The segmented crystals are around 20 nm thick, and thus have low electron and hole recombination, contributing to enhanced PCA.

We believe that the high growth rate of 160 μm/h that was achieved during the peak of the injection pulse plays a central role in dendrite generation. High growth rates have been shown to produce columnar structure in thick titania films^43^, but there are only a few reports of dendrites or feathered nanostructures of CVD TiO_2_. Takahashi reported up to 60 μm thick films with growth rates ranging from 10 to 85 μm/h using CVD of isopropyl titanate with O_2_ reactant in N_2_ carrier gas^25^. The reported microstructure was columnar anatase and dendritic rutile, but no observation of nano-plates was made. Laser Chemical Vapor Deposition (LCVD) produced feather-like morphologies when growing different ceramics at high temperature, including TiO_2_ films^26,44^. Growth rate of 2500 µm/h was estimated for the feathered TiO_2_ material with (101) texture. None of the feathered nanostructure TiO_2_ films have been reported to contain carbon and all of them were processed with O_2_ reactant gas. The previously reported feathered structures also had very large crystals that did not form a solid coating layer. Feathered nanostructure was also observed in columnar Yttria-Stabilized Zirconia (YSZ) coatings processed by Electron Beam Physical Vapor Deposition (EB-PVD) with remarkably high growth rate ranging from 120 to 630 µm/h and thickness up to 300 μm^45^.

The wear-debris of NsARC could include nanoparticles, and this is an area of on-going research. Future studies will investigate the durability in more detail, and the role of the nano-plates, dendritic nanocrystals and carbon in scratch resistance and adhesion. Future work will also investigate the range of deposition parameters and the effects on the details of the nanostructure, composition and the PCA.

In conclusion, nanoengineering of high surface area structures in a solid coating material through CVD processing represents a significant advance in the potential for manufacturing highly photoactive TiO_2_ coating materials on practical surfaces without the inherent issues of manufacturing, handling and incorporating nanoparticles into a coating.

## Methods

### Substrate preparation and film processing

All NsARC films were deposited on 25 × 25 × 1 mm^3^ stainless steel (type 304) and fused silica substrates by pp-MOCVD using a custom-built reactor^42^. The periodic direct liquid injection process and equipment used to manufacture this material has been described previously^22^. The pump-down base pressure was 100 Pa. Liquid precursor solution with 5:95 TTIP to toluene molar ratio was used. Stainless steel substrates were cleaned by abrading with 400 grit sandpaper, followed by ultrasonication in a silicon-free detergent and water solution, flushing with deionized water, and drying with an air gun before loading into the pp-MOCVD chamber and proceeding with 30 min bake-out. TTIP (titanium tetraisopropoxide) from Sigma Aldrich was mixed with dry, high performance liquid chromatography (HPLC) grade toluene in a dry glovebox and loaded into an ampoule. Deposition temperatures of 500 °C for stainless and 525 °C for fused silica substrates were accomplished by induction coil heater. Precursor pulse volume was 500 µl, pulse interval was 6–8 s and number of pulses was 735–750. Typical coating thickness was 10–11 μm.

### SEM Characterization

The SEMs used were JEOL 7000 F and Zeiss Ultra 55 field emission scanning electron microscope. The films deposited on stainless steel were imaged as grown. Films on fused silica were sputtered with chromium prior to imaging. Fracture surface cross-sections were prepared by scoring and snapping.

### TEM Observations

Two different techniques of transmission electron microscopy (TEM) were used. JEOL 2100 F and Philips CM-200 at 200 kV with a lanthanum hexaboride (LaB_6_) filament. The films were removed from the substrate by diamond tip scribe and transferred onto a copper grid with formvar support film and coated with carbon film prior to imaging.

### ACOM also known as ASTAR^TM^

Thin cross-sections of the coating were prepared by a Zeiss NVision 40 FIB-SEM equipped with a MEB GEMINI column using a Schottky type field emission gun. The equipment also has scanning transmission electron microscopy capability and a FIB SIINT ZETA column that provides Ga^+^ ions. Thin film coating of 0.5 μm Pt was deposited *in situ* using a metal organic precursor to extract a 2 × 12 μm^2^ cross-section of the coating. Another layer of Pt was deposited on the extracted sample to avoid twisting of the sample due to electromagnetic forces. Other local *in situ* carbon depositions were performed to weld the cross-section to the tip of a positioning needle.

The automated crystal orientation and phase mapping (ACOM)^29^ technique also referred to as ASTAR^TM^ was used to characterize the different phases and orientations in the NsARC films. Selected Area Electron Diffraction (SAED) patterns were obtained using a JEOL 2100 F TEM at 200 kV with a precession angle of 1.2° and a step size of 10 nm. The ASTAR^TM^ matching procedure uses TiO_2_ IUCR crystallography data:

Anatase: tetragonal with space group I 4_1_/a m d (No. 141) and lattice parameters *a* = *b* = 3.786 Å and *c* = 9.519 Å, α = β = γ = 90°

Rutile: tetragonal with space group P 4_2 _/m n m (No. 136) and lattice parameters *a* = *b* = 4.595 Å and *c* = 2.959 Å, α = β = γ = 90°.

### X-ray Diffraction

X-ray diffraction (XRD) was used for phase identification and texture analysis. This was carried out on an *Agilent SuperNova*, Dual, Cu at zero, Atlas diffractometer using Cu Kα (λ = 1.5418 Å) radiation. Samples were mounted vertically with the surface close to parallel to the ϕ-axis, and aligned so that 0° in ϕ corresponded to the sample also being parallel to the beam. The sample was rotated from 10° to 80° in ω with the detector positioned to collect data from 0° to 95° in 2θ in a single position with two correlated frames of 300 s. Post collection, the frame was integrated over a 5° strip corresponding to a pseudo θ–2θ diffractometer. To correct for slight differences in the mounting alignment, the integration angle in γ was calculated as the average of two off axis, symmetry related peaks at 2θ = 25°. 2θ angles were corrected using P25 standard (Sigma Aldrich).

### Surface Enhanced Raman Spectroscopy

Surface enhanced Raman spectra for the NsARC films were obtained using Jobin-Yvon LabRam single stage spectrometer with a 400 μW powered 514 nm Ar-ion laser. The depth resolution was around 15 nm. The carbon content was analyzed by isolating the peaks between 1000 and 2500 cm^−1^. Spectral peaks were identified using Horiba dataset^46^ and Ferrari *et al*.^47^.

### X-ray Photoelectron Spectroscopy

Surface analysis was performed by XPS using a XR3E2 apparatus from Vacuum Generator employing an Mg Kα source (1253.6 eV). The X-ray source was operated at 15 kV for a current of 20 mA. The sample preparation was carried out in order to ensure the results were representative of the depth of the film. Before data collection, the samples were stored for 12 hours in an ultrahigh vacuum chamber. Samples were etched with a 50 W Ar plasma for 10 minutes just prior to analysis in order to remove remaining surface contamination. Photoelectrons were collected by a hemispherical analyzer at a constant take-off angle of 90°. Spectra were calibrated with respect to the C1s peak at 285 eV. Deconvolution of the peaks was performed using a 10% Lorentzian/Gaussian function after subtraction of the background by the Shirley method. The peaks were identified using NIST database^48,49^.

### Photocatalytic antimicrobial activity measurements

Antimicrobial testing of NsARC coated samples used the ISO 274 47:2009 standard test method^50^. *E*. *coli* ATCC 8739[AMA1] was grown at 37 °C to saturation, approximately 10^9^ colony forming units (cfu ml^−1^) in nutrient broth [AMA2] using a shaker platform for aeration. Bacteria were pelleted by centrifugation and washed using PBS. This was repeated three times before a final resuspension of the bacteria. Samples were then diluted to approximately 10^7^ cfu ml^−1^ in PBS [AMA3]. Test (NsARC) and control (glass slide) samples were sterilized using 70% ethanol. NsARC was stored in the dark for >48 hours to discharge. Test and control samples were then placed in sterile petri dishes on a sterile glass slide separating the sample from wet sterile filter paper at the bottom of the petri dish. 50 μl of the bacterial suspension was then aseptically placed on top of the samples and immediately covered with cover slip that had been sterilized with 70% ethanol. Samples (in triplicate) were then exposed to UV (365 nm wavelength), visible light with intensity 1500 lumens, or kept in the dark for periods of 4 hours. After exposure, bacteria were recovered in 1.95 ml Tryptic Soy Broth [AMA4,5]. Then 100 μl of the recovered bacteria was serially diluted and transferred on to the surface of Tryptic Soy Agar in a petri dish in 10 μl spots. Plates were incubated at 37 °C for 18–24 hours before colonies were counted.

The cfu ml^−1^ values were converted to efficiency of plating (EOPs).

$${\rm{EOP}}=\frac{{\rm{Titre}}\,{\rm{of}}\,{\rm{plates}}\,{\rm{after}}\,{\rm{treatment}}(\frac{{\rm{cfu}}}{{\rm{ml}}}of\,treatments)\,for\,4\,hours}{Initial\,titre\,from\,control\,plates(\frac{cfu}{ml})\,}$$

The cfu ml^−1^ are representative averages of 3 technical replicates. Each experiment was repeated 3 times independently giving n = 3 for statistical tests. In the case of measured cfu ml^−1^ dropping below the detection limit, the detection limit itself was used as the sample value for statistical analyses.

### Antimicrobial testing materials

AMA1 *Escherichia coli* (Migula) Castellani and Chalmers ATCC ® 8739&trad. (n.d.). Retrieved 21 September 2017, from https://www.atcc.org/en/Standards/Quality_Control_Strains/Media_testing/8739.aspx

AMA2 CM0001, Nutrient Broth | Oxoid - Product Detail. (n.d.). Retrieved 21 September 2017, from http://www.oxoid.com/UK/blue/prod_detail/prod_detail.asp?pr = CM0001&cat = &sec = 1

AMA3 http://www.protocolsonline.com/recipes/phosphate-buffered-saline-pbs/

AMA4 CM0001, Nutrient Broth | Oxoid - Product Detail. (n.d.). Retrieved 21 September 2017, from http://www.oxoid.com/UK/blue/prod_detail/prod_detail.asp?pr = CM0001&cat = &sec = 1

AMA5 CM0129, Tryptone Soya Broth (Soybean Casein Digest Medium USP) | Oxoid - Product Detail. (n.d.). Retrieved 21 September 2017, from http://www.oxoid.com/UK/blue/prod_detail/prod_detail.asp?pr = CM0129

## Supplementary information

Supplementary Information

## Data Availability

The datasets generated during and/or analysed during the current study are available from the corresponding author on reasonable request.
