# Supplementary material for: Nanostructured TiO2 anatase-rutile-carbon solid coating with visible light antimicrobial activity
Source: Sci Rep. 2019 Feb 13;9:1883. doi: 10.1038/s41598-018-38291-y (PMC6374394; doi:10.1038/s41598-018-38291-y)
Supplement: Supplementary file 1 — Supplementary Information [file 41598_2018_38291_MOESM1_ESM.pdf]

Nanostructured TiO_2 anatase-rutile-carbon solid coating with visible light antimicrobial activity

Susan P. Krumdieck[1*], Raphaël Boichot[2], Rukmini Gorthy[1], Johann G. Land[1], Sabine Lay[2], Aleksandra J. Gardecka[1], Matthew I. J. Polson[3], Alibe Wasa[4], Jack E. Aitken[4], Jack A. Heinemann[4], Gilles Renou[2], Grégory Berthomé[2], Frédéric Charlot[2], Thierry Encinas[2], Muriel Braccini[2], Catherine M. Bishop[1]

SUPPLEMENTARY MATERIAL

Durability of NsARC Coatings

The adhesion of NsARC coatings to 25×25×1 mm^3 stainless steel substrates was analyzed according to the ASTM standard (D 3359) cross-cut tape test using a Sctoch^{TM} 3M semi-transparent tape. The test was performed on five identical specimens and the results were classified as grade 5B, the highest level of adhesion. Fig. S1 (a) shows an NSARC coating on stainless steel (type 304) substrate that has been subjected to Scotch tape test with result of 0% of the area removed.

The fracture strength, energy release rate of propagating cracks, and adhesion strength of the film were evaluated using a 4-point bending test[S1,S2]. Upon bending, the cracking and delamination of the film is recorded and related to the film durability properties. Stainless steel substrates 100mm x 10mm x 1mm were coated with NsARC using the same deposition conditions described herein. The bending force was applied through stiff lever arms glued to the film surface. However, the standard 4-point bending adhesion test of two samples resulted in delamination of the glue from the bending arm, with no damage or delamination of the coating. Thus, the adhesive force of the coating on stainless steel is superior to 50 J/m^2, which is the strength of the adhesive.

NsARC coated SS substrates (75×25×1 mm^3) were subjected to 3-point bending using an MTS Criterion (C43.104). The samples were bent to approximately 60° with a load of 2.5 kN. The bend section does not show any delamination of the coating and is bordered by distinct intact lines caused by the compression of the bending roller bars (Fig. S1 b). This result is a clear indication of the robustness and durability of the NsARC coatings. This robustness could possibly be used in manufacturing. However, the pp-MOCVD process can uniformly coat 3-D shapes[S3], so the coating would be applied in general on products after forming processes are completed.

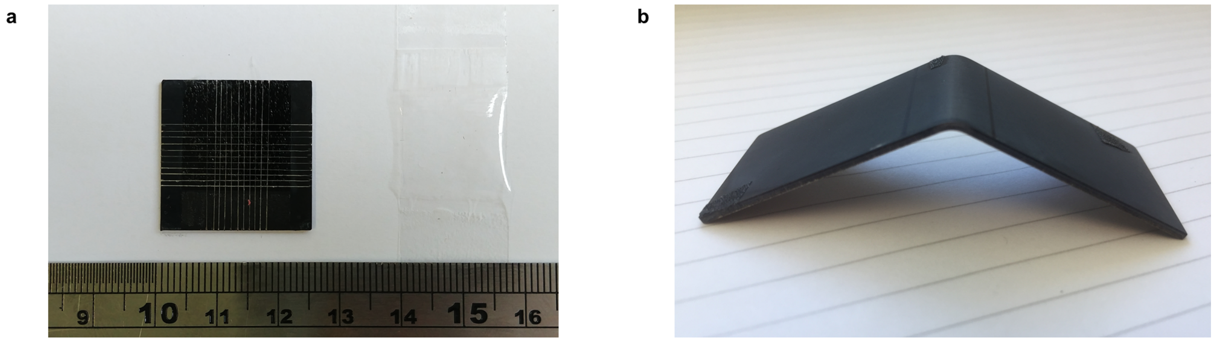

Figure S1. Durability tests demonstrating the robust nature of the NsARC coatings. (a) Scotch tape test showing no delamination, (b) 3-point bending test showing no delamination of the black coating from the stainless steel substrate.

As-deposited and annealed coatings on fused silica

NsARC samples were annealed at 500 °C in air for 2 hours to investigate the state of carbon in the material. Fig. S2 shows photographs of a 10 µm thick NsARC coated substrate of fused silica before and after annealing. The as deposited sample is remarkably black and completely opaque. Post-annealing, the color changes from black to white, indicating the oxidation and removal of the carbon in the coating. The white annealed sample is translucent and appears to be macroscopically similar to commercial P25. This degree of whiteness and opacity in a solid TiO_2 material, indicating the presence of many surfaces that scatter and diffuse light.

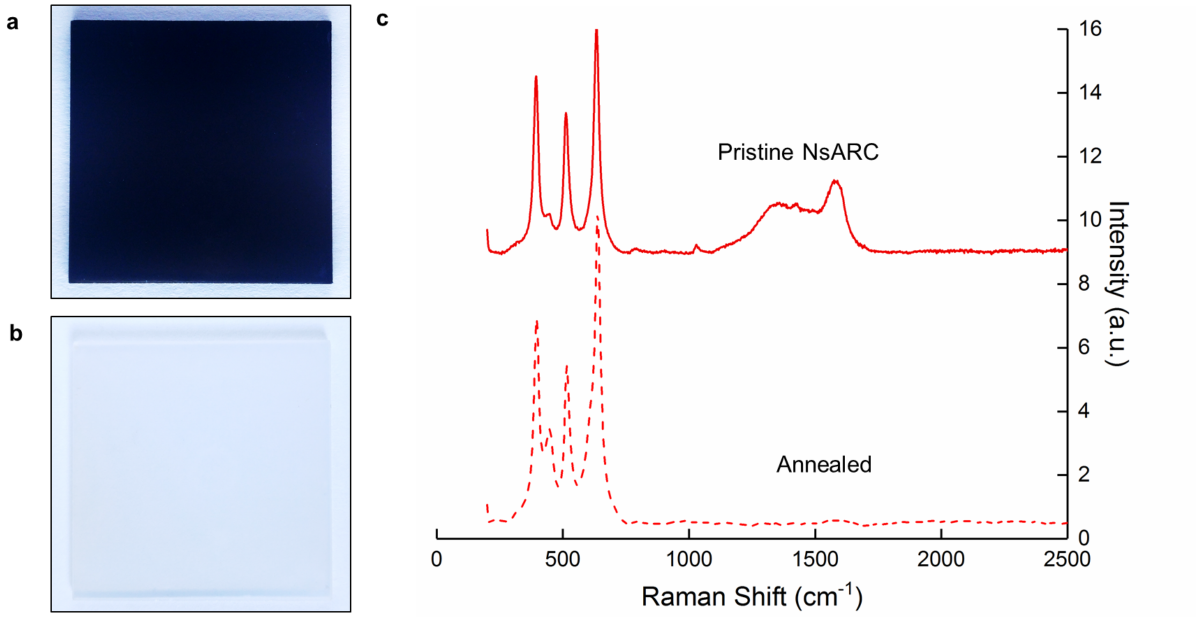

Figure S2. Analysis of the NsARC coating on fused silica substrate before and after annealing. (a) Photograph as-deposited. (b) Photograph after annealing. (c) Raman spectra of as-deposited and annealed NsARC coatings.

Raman analysis of the annealed sample showed no carbon peaks in the spectrum (Fig. S2 c). The Raman spectra exhibited a significant G band around 1600 cm^{-1} and shorter but broader D band around 1450 cm^{-1} (Fig. S2). The D band refers to disorder in a graphitic structure due to the breathing modes of rings while G band refers to the C-C bond stretching and is common to all sp^2 type carbon materials. The deconvolution resulted in five distinct peaks which were identified based on Horiba dataset[46] and Ferrari *et al*[47]. The peaks at 1499 cm^{-1} and 1576 cm^{-1} correspond to the presence of aromatic rings. The peak at 1615 cm^{-1} corresponds to the presence of sp^2 bonds between carbon atoms. Moreover, the spectrum exhibited a weak peak at 1435 cm^{-1} corresponding to single bonds between carbon and hydrogen atoms, revealing the presence of polymer-like carbon chains. Finally, the first peak at 1354 cm^{-1} indicated the presence of carboxylate.

Crystallographic analysis of nanostructures

The nanostructure of the NsARC features plated segmentation of the anatase columnar crystals and rough dendritic branching of the rutile columns (Fig. S3). The TEM images were acquired by scraping off NsARC films from the substrate and crushing them. SAED patterns obtained from different areas of a single anatase column indicated that all the nanoplates have the same orientation. This sample preparation technique appears to have disintegrated the dendritic structures and thus similar analysis could not be performed on rutile columns.

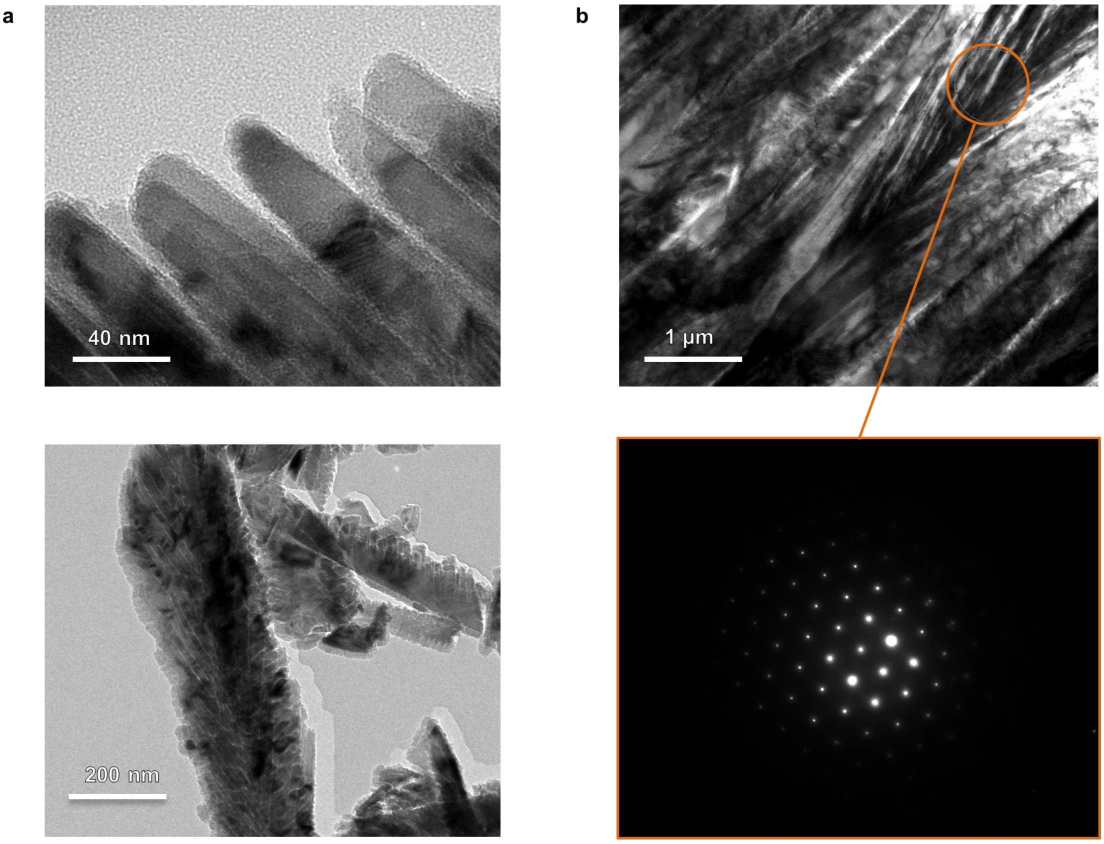

Figure S3. Crystallographic analysis of nanostructures. (a) TEM micrographs of sample debris showing the plate-like features of an anatase *mille-feuille* crystal, (b) High magnification SEM image showing the nanoplates on rutile *strobili* (c) FIB cross section with SAD of numerous anatase plates showing single-crystal pattern.

FACTSAGE Thermodynamic Modelling

Liquid precursor is directly injected into the deposition chamber via an ultrasonic atomizer. The droplet evaporation and vapor expansion occurs in under 100 ms. Flash evaporation of the precursor generates a steep pressure pulse to 890 Pa followed by pump down to 100 Pa over the ensuing 6-10 second pulse cycle[S4]. Nearly all of the precursor flux to the surface would occur within 340 ms according to modelling of the molecular dynamics[S5] (Fig. S4). The thermodynamic stability of a mixture of 5:95 TTIP and toluene vapor was assessed by entropy maximization with Factsage 7.1 software and FACT database[S6]. The calculations covered a pressure range of 100 to 500 Pa and temperature of 500 and 525°C. Calculations considered all species of the Ti-C-H-O system referenced in the database, limiting the maximum number of carbon for organic compounds to six. All phases were considered. Calculations used two constraints: fixed number of atoms and fixed pressure (varying final volume). Results of the solid carbon molar fraction at equilibrium for the pressures experienced during the pressure pulse are shown in Fig. S4.

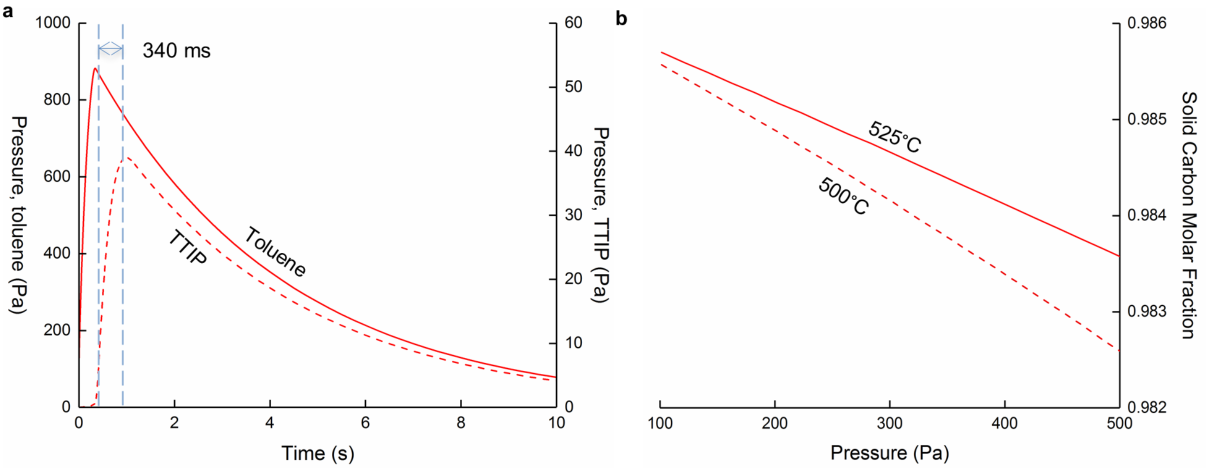

Figure S4. Results of modelling. (a) Vapor partial pressure of 5:95 TTIP and toluene vapor during droplet evaporation and pump-down. (b) Solid molar concentration with temperature and pressure at final thermodynamic equilibrium.

Surface Area Calculation

The specific surface area for powders is usually measured using the BET (Brunauer, Emmett and Teller) method. However, thin films and coatings cannot be effectively analyzed using the BET method. The specific surface area of NsARC was measured by analyzing the adsorption of Methylene Blue Dye (MBD) into the microstructure. Adsorption tests were performed using a 7.34 mg/L MBD solution for 9.5 µm thick NsARC samples on fused silica substrates and blank fused silica substrates. Samples were soaked in 40 mL solution and kept in the dark for 90 minutes. The test was repeated 5 times. The decrease in MBD concentration was observed using a *Cintra 4040* UV-Vis spectrophotometer. Absorbance was correlated to concentration using a calibration curve. The observed loss in MBD after 90

minutes of adsorption in the dark was calculated to be 0.11 +/- 0.03 μmol. The blank substrate did not show any decrease in concentration of the solution.

Adsorption of MBD on TiO_2 powder has been reported in the literature for TX titania[S7]. TX titania has a Brunauer-Emmett-Teller (BET) surface area of 10 m^2/g. It was reported that TX titania adsorbed $1.6x10^{-5}$ mol MB/g. Using the BET surface area of TX titania powder, the MBD adsorption on TiO_2 is calculated to be $1.6x10^{-6}$ mol MB/m^2. Using this equilibrium adsorption value, the specific surface area for NsARC is calculated as 80-140 m^2/m^2 of substrate and $8x10^6$-$15x10^6$ m^2/m^3 for the coating material. As a reference, commercially available P25 has a granular density of 4.26 g/mL with a reported surface area of 35-65 m^2/g of powder, giving a specific area in the range of $150x10^6$ to $280x10^6$ m^2/m^3. Thus, the NsARC specific surface area is about ten times lower than P25 powder, and 100 times higher than a solid, non-porous coating of TiO_2 such as the commercially available Pilkington Activ™ coating.

Antimicrobial Testing

The antimicrobial activity was analyzed according to international standard ISO 27447:2009 using *Escherichia coli*. Test film surface area was 525 mm^2 of NsARC on stainless steel. The specific conditions used for the testing are:

- LED Light source conforming to the standard
- The test period was 4 hours
- Glass was used as the control surface

Photocatalytically produced ROS are the primary cause of lethality, but surface topography could play a role. Topography mediated surface-bacterial interactions is an emerging field of research[S8]. The *E coli* bacteria used to assess antimicrobial activity are roughly 1-2 microns long. This is on the order of the shape scale of the anatase and rutile crystals at the surface (Fig. S5 a). The bacteria are not likely to be trapped in the porous structure of the material as they are larger than the pore size between the columns (Fig. S5 b).

The recent discovery of bactericidal properties of insect wings being related to the nanostructured array of pillars may have relevance to the NsARC topography playing a role in the *E. coli* killing in the dark[S9]. Biofilm formation can be inhibited by hydrophilic surfaces composed of pillars with dimensions less than the dimeter of a single bacterium[S10]. The Surface nano-topography of the NsARC coating is composed of microcolumns and is hydrophilic compared to bare stainless (Fig. S5 c). However, the antibacterial testing method used in this work would not be affected by the surface wettability.

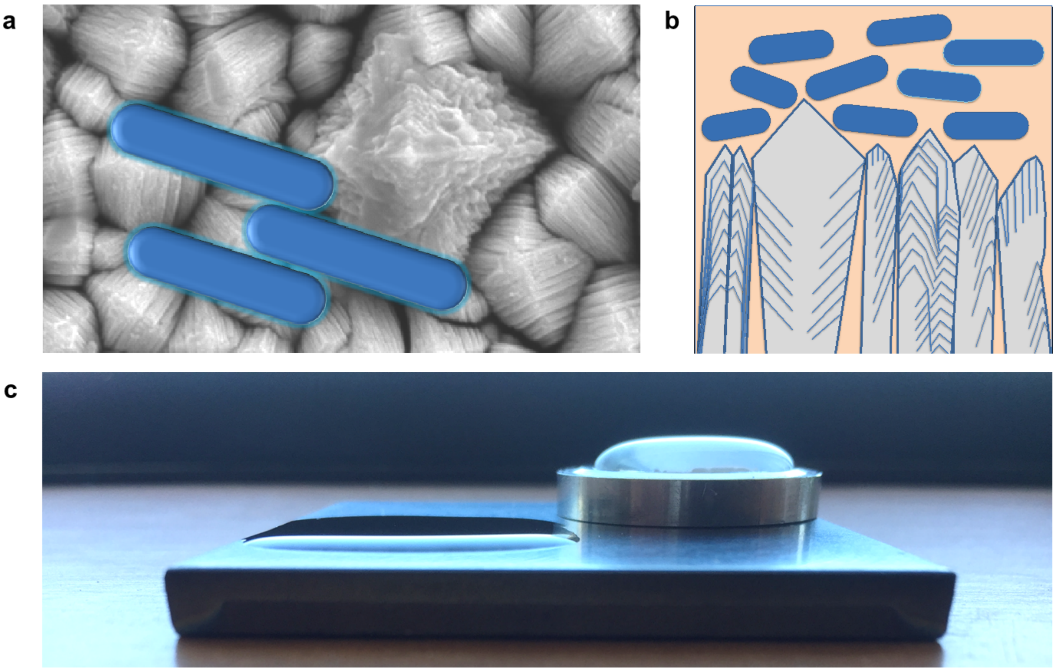

Figure S5. Bacteria interaction with NsARC. (a) Surface morphology of NsARC with reference scale representation of *E coli.* (b) Scale drawing of NsARC crystals and local porosity compared to bacteria in aqueous suspension. (c) Comparison of water wetting of uncoated stainless steel (round) and NsARC coated stainless steel (square).

Supplementary References

S1. Charalambides, P. G., Lund, J., Evans, A. G., & McMeeking, R. M. A test specimen for determining the fracture resistance of biomaterial interfaces. *J. of Appl. Mech.*, 56, 77 (1989).

S2. Hofinger, I., Oechsner, M., Bahr, H., & Swain, M. V. Modified four-point bending specimen for determining the interface fracture energy for thin, brittle layers. *Int. J. of Fract.*, 92, 213–220 (1998).

S3. Krumdieck, S., Gorthy, R., Gardecka, A.J., Lee, D., Miya, S., Davies Talwar, S., Polson, M. I. J., Bishop, C. Characterization of photocatalytic, wetting and optical properties of TiO2 thin films and demonstration of uniform coating on a 3-D surface in the mass transport controlled regime, *Surf and Coat. Technol.*, 326(B), 402-410 (2017).

S4. Boichot, R. & Krumdieck, S. Numerical Modeling of the Droplet Vaporization for Design and Operation of Liquid-pulsed CVD. *Chem. Vapor Depos.* 21, 375-384 (2015).

S5. Cave, H. M., Krumdieck, S. P. & Jermy, M. C. Development of a model for high precursor conversion efficiency pulsed-pressure chemical vapor deposition (PP-CVD) processing. *Chem. Eng. J.* 135, 120-128 (2008).

S6. FACTSAGE 7.1 Database. CRCT-Thermfact Inc. & GTT-Technologies http://www.factsage.com/ (2017).

S7. Fetterolf, M. L., Patel, H. V. & Jennings, J. M. Adsorption of methylene blue and acid blue 40 on titania from aqueous solution. *J. Chem. Eng. Data* 48, 831-835 (2003).

S8. Hasan, J. & Chatterjee, K. Recent advances in engineering topography mediated antibacterial surfaces. *Nanoscale* 7, 15568-15575 (2015).

S9. Ivanova, E. P., Hasan, J., Webb, H.K., Truong, V.K., Watson, G.S., Watson, J.A., Baulin, V.A., Pogodin, S., Wang, J.Y., Tobin, M.J., Lobbe, C. Crawford, R.J. Natural bactericidal surfaces: mechanical rupture of *Pseudomonas aeruginosa* cells by cicada wings. *Small*, 8(16) 2489-2494 (2012).
S10. Xu, L-C, Siedlecki, C.A. *Staphylococcus epidermidis* adhesion on hydrophobic and hydrophilic textured biomaterial surfaces. *Biomed. Mater.* 9, 035003 (2014).
